# Supplementary material for: Examining the infographic design instructional process in terms of prospective mathematics teachers’ infographic design proficiency, self‑efficacy, and abilities in evaluating student errors: A model proposal
Source: PLoS One. 2026 Apr 17;21(4):e0341380. doi: 10.1371/journal.pone.0341380 (PMC13089900; doi:10.1371/journal.pone.0341380)
Supplement: S1 Appendix — (DOCX) [file pone.0341380.s001.docx]

**Appendix 1. Infographic design self-efficacy scale (IDSES).**

| **No** | **ITEMS** | **Totally Inadequate** | **Inadequate** | **Undecided** | **Adequate** | **Totally Adequate** |
| --- | --- | --- | --- | --- | --- | --- |
| ***Competencies in Designing Infographics in Digital Environments*** | | | | | | |
| Via a graphic editor; | | | | | | |
| **1** | Able to draw a shape with a vector tool |  |  |  |  |  |
| **2** | Able to assemble objects |  |  |  |  |  |
| **3** | Able to color objects |  |  |  |  |  |
| **4** | Able to apply effects to inserted objects |  |  |  |  |  |
| **5** | Able to change the color of written text |  |  |  |  |  |
| **6** | Able to apply effects to written text |  |  |  |  |  |
| **7** | Able to use stock images |  |  |  |  |  |
| **8** | Able to edit stock images |  |  |  |  |  |
| **9** | Able to resize objects |  |  |  |  |  |
| **10** | Able to create new work pages |  |  |  |  |  |
| **11** | Able to perform erasing operations on objects |  |  |  |  |  |
| ***Competencies in Designing Infographics According to Visual Design Principles and Elements*** | | | | | | |
| **12** | Able to align elements in accordance with visual design principles |  |  |  |  |  |
| **13** | Able to apply the principle of proximity to express relationships among elements |  |  |  |  |  |
| **14** | Able to employ the principle of direction to guide the viewer’s attention from one point to another |  |  |  |  |  |
| **15** | Able to establish a figure–ground relationship in a contrasting structure for accurate reading and perception of content |  |  |  |  |  |
| **16** | Able to use colors in keeping with visual design principle |  |  |  |  |  |
| **17** | Able to achieve overall unity in line with visual design principles |  |  |  |  |  |
| **18** | Able to add three dimensional effects to objects |  |  |  |  |  |
| **19** | Able to add three dimensional effects to text |  |  |  |  |  |
| **20** | Able to adjust image brightness to enhance visual perception |  |  |  |  |  |
| **Competencies for Using Infographics in the Instructional Process** | | | | | | |
| **21** | Able to select infographics appropriate to course content |  |  |  |  |  |
| **22** | Able to select infographics aligned with course objectives |  |  |  |  |  |
| **23** | Able to employ infographics that concretize abstract information |  |  |  |  |  |
| **24** | Able to employ infographics that simplify complex information |  |  |  |  |  |
| **25** | Able to use infographics that facilitate recall of prior knowledge |  |  |  |  |  |
| **26** | Able to use infographics that display relationships among concepts and elements |  |  |  |  |  |
| **27** | Able to use infographics that enhance learners’ thinking skills |  |  |  |  |  |
| **28** | Able to draw learners’ attention by means of engaging infographics |  |  |  |  |  |
| **29** | Able to employ infographics that learners can readily comprehend |  |  |  |  |  |
| **30** | Able to employ infographics unlikely to cause misinterpretation of content |  |  |  |  |  |
| **31** | Able to use infographics that provide opportunities for practice and review |  |  |  |  |  |
| **32** | Able to select infographics appropriate to the learners’ age level |  |  |  |  |  |
| **33** | Able to select infographics appropriate to the learners’ proficiency level |  |  |  |  |  |
| **34** | Able to select infographics prepared to match the learners’ cognitive readiness |  |  |  |  |  |
| **35** | Able to employ infographics with up-to-date content |  |  |  |  |  |
