# Supplementary material for: Examining the infographic design instructional process in terms of prospective mathematics teachers’ infographic design proficiency, self‑efficacy, and abilities in evaluating student errors: A model proposal
Source: PLoS One. 2026 Apr 17;21(4):e0341380. doi: 10.1371/journal.pone.0341380 (PMC13089900; doi:10.1371/journal.pone.0341380)
Supplement: S2 Appendix — (DOCX) [file pone.0341380.s002.docx]

**Appendix 2. Sample items from the error evaluation pre-test and post-test.**

| Pre Test  Question 1 (Koparan, 2012).  Muğla Vacation | Question | A researcher wishes to determine the province most frequently chosen for holidays in Türkiye. The researcher goes to a shopping mall in Muğla and, at random, asks 10 store employees and customers which province they most often visit for holidays. Using these responses, the researcher identifies the province most preferred for vacations in Türkiye.  (*) What do you think about the people the researcher selected?  (**) If you were the researcher, whom would you choose for your study? Explain your reason. |
| --- | --- | --- |
|  | Middle school student’s answer | *(*) It makes perfect sense that the researcher went to a store in Muğla. Since it is a resort area, asking people there is logical.*  *(**) I would ask employees in a travel agency because they know this business better.* |
| Post Test Question 1  (Watson, 2006, as cited in Topan, 2019)  Charity Campaign | Question | Students at a middle school plan to run a charity campaign for a nearby village school. First, they want to gauge their own schoolmates’ willingness to participate, so they decide to conduct a survey. The school has 800 students, 200 in each grade from 5 through 8.  (*) If you were conducting this study, how would you select the students to survey? Explain your reason.  (**) Mehmet wrote the names of all 800 students on separate slips of paper, placed them in a bag, and drew 80 names. What do you think of Mehmet’s research method? |
|  | Middle school student’s answer | *(*) If I were collecting donations, I would choose well-off students because they can help more.*  *(**) That is very wrong. Some of the 80 students may not wish to join the campaign.* |
| Pre Test  Question 3  (Topan, 2019)  Football Match Game on Computer | Question | Enes is assembling his team for a football match in a computer game. For the final spot he must choose between Burak and Cenk. He reviews the number of goals each scored in their recent matches:  Burak: 3 6 2 3 1  Cenk: 4 3 1 5 3 2  Which player should Enes select? Explain your reasoning. |
|  | Middle school student’s answer | *Burak scored 15 goals, Cenk scored 18. Enes should choose Cenk.* |
| Post Test Question 3  (Topan, 2019)  Umut–Sevgi Middle Schools | Question | A middle school chess tournament is being held in Samsun. Each school sends one student. Umut and Sevgi Middle Schools have reached the semifinals. Their scores during the tournament are listed below. Which school do you think will reach the final? Explain your reasoning.  Sevgi Middle School: 6 9 3 4 1 7 Umut Middle School: 8 2 3 6 6 |
|  | Middle school student’s answer | *Sevgi School, because its scores are higher.* |
