# Supplementary material for: Examining the infographic design instructional process in terms of prospective mathematics teachers’ infographic design proficiency, self‑efficacy, and abilities in evaluating student errors: A model proposal
Source: PLoS One. 2026 Apr 17;21(4):e0341380. doi: 10.1371/journal.pone.0341380 (PMC13089900; doi:10.1371/journal.pone.0341380)
Supplement: S3 Appendix — (DOCX) [file pone.0341380.s003.docx]

**Appendix 3. Sample excerpts illustrating the change and improved quality of the prospective teachers’ corrective suggestions.**

PT40 (EEPrT-Muğla Vacation): *“I would ask, ‘Are people working at a travel agency sufficient for determining the province most preferred for holidays in Türkiye?’ If necessary, I would use a map of Türkiye to show students the analysis.*

PT40 (EEPoT - Charity Campaign): *To help the student see the error, I would explain the basics: What is data collection? What are the population and the sample? How is a sample selected, and why is random selection important? I would pose questions that prompt students to investigate data collection and interpretation, using a combination of questioning and discussion techniques.*

PT33 (EEPrT -Ice Age 5 Movie): *We may assume the student does not know how to interpret a pie chart. We could ask the student to sum all the values and report the total. If the student still notices nothing, we might conclude that the student has difficulties in other areas as well.*

PT33 (EEPoT - Vote Distribution): *I would ask the student how a pie chart is constructed, read, and interpreted, posing questions that require interpreting the chart. I would explain the importance of ratio and percentage in that interpretation.*

PT27 (EEPrT - Ice Age 5 Movie): *It is clear the student does not grasp the criteria and logic of a pie chart, the student simply filled in an answer space. Therefore, the pie chart concept should be retaught.*

PT27 (EEPoT – Vote Distribution): *“Students failed to make the connection because they do not realize that pie charts relate to angles… I would ask, ‘How and on what basis do you construct a pie chart? If Party Y has A voters, how many does Party Z have? How will you decide?’ I would then pose further questions on ratio and proportion, representative display, graphs, and percentage calculation, fostering classroom discussion. Through cooperative learning, students would recognize their errors and master the topic.*
