# Supplementary material for: Examining the infographic design instructional process in terms of prospective mathematics teachers’ infographic design proficiency, self‑efficacy, and abilities in evaluating student errors: A model proposal
Source: PLoS One. 2026 Apr 17;21(4):e0341380. doi: 10.1371/journal.pone.0341380 (PMC13089900; doi:10.1371/journal.pone.0341380)
Supplement: S4 Appendix — (DOCX) [file pone.0341380.s004.docx]

**Appendix 4. Detailed answers to the EEPrT / EEPoT items and sample instructional explanations provided by the prospective teachers.**

In the questions titled Ice Age 5 Film and Vote Distributions, which require assessing whether the graph type chosen is appropriate and correctly drawn, the middle school student provides a superficial and incorrect answer. In the pie chart, the student fails to notice that the sum of the sectors exceeds 100 percent, the 55 percent slice occupies more than half of the circle, and the graph’s title does not correspond to the displayed data. Prospective teachers are expected to detect these inconsistencies and errors and to state that the student’s answer is incorrect. Similarly, in the Vote Distributions item, where election percentages for two different years are presented visually without numerical labels, the student is unable to interpret the graphs correctly. The primary reason for the error is the student’s lack of knowledge of fundamental mathematical concepts such as ratio and percentage, and the inability to relate those concepts to the pie chart, in other words, the student cannot determine proportional relationships among the data. Therefore, prospective teachers should propose solutions by posing questions that prompt the student to recognize the error and reconsider the answer. For example, a prospective teacher might ask: *“What is a pie chart? What kinds of data should we represent with a pie chart?” “What is the total of all the sectors in this chart? What does that total mean?” “Do the percentages given for each sector match the sizes shown? Why or why not?” “What do you think about the chart’s title? Could you comment on it?”* Essentially, the prospective teacher needs to explain how the student is reasoning during interpretation, ask questions that help the student identify the mistake, and recommend instructional methods or strategies to resolve the difficulty. Questions such as *“How and on what basis does a sector’s size change in a pie chart?” or “If the vote percentages or central angles of the X and Z sectors in both charts were provided, how would your interpretation change?”* can guide the student to see the error. Additional strategies might include using computer assisted mathematics tools such as GeoGebra, having students redraw the given pie charts digitally and discuss the key considerations in that process, and asking students to research real vote share data from different years, represent them as pie charts, interpret them, and explain their solutions.

In the questions entitled *Football Match Game on Computer* and *Umut–Sevgi Middle Schools*, which require calculating the mean and range of two different data sets, prospective teachers are expected to offer correct remedial suggestions addressing the error the student makes while comparing the two sets using measures of central tendency and dispersion. In *Football Match Game on Computer*, the student adds the number of goals each player scored to obtain a total and, because this total is higher, chooses Cenk. However, Burak and Cenk have played a different number of matches, something the student overlooks, erroneously assuming that the total number of goals is sufficient. The student should have calculated the mean for both data sets, noticed the unequal number of matches, and decided using the range. Thus, prospective teachers should state that the student erred because the match counts differ. The error may stem from a lack of foundational knowledge regarding measures such as mean, mode, median, and range. Questions that might help the student recognize the error include: *How many matches has each player played? If Burak played one more match, how many goals might he score? Is it appropriate to decide solely on total goals?* Similarly, the *Umut–Sevgi Middle Schools* item requires finding the mean and range and comparing two data sets. The student should realize that the two schools have the same mean score and then decide using the median and range, however, the student responds incorrectly. A prospective teacher might explain the error as follows: *the student focused on the total score without using the mean and range, concluding incorrectly that Sevgi should advance because its total is higher. Yet the schools played a different number of matches, so the student should have employed the mean and range.* Questions to reveal the error could include*: How many matches did each school play? If Umut played one more match, how many points might it earn? Is it appropriate to decide solely on total points? Why do you think Sevgi should advance? Does the number of matches affect the decision? Is summing points sufficient? Why? How would you decide?* Prospective teachers might attribute the error to the student’s failure to understand the mean concept or how outliers influence the mean, i.e., a knowledge gap about the effect of extreme values. Possible remedial suggestions include asking, *What is the mean? What are mode, median, and range? When comparing two data sets or selecting one, which measure would you consider first?* Prospective teachers could also assign activities focusing on the conceptual rather than procedural aspects of mean, mode, median, and range.

In the *Student Counts Table* and *Foreign Language Instruction* items, students are asked to select, draw, and interpret an appropriate type of graph. In the *Student Counts Table* response, the student makes no errors in scaling, labeling the axes, or transferring the required data to the graph but chooses an inappropriate graph type. In the *Foreign Language Instruction* item, the student draws a line graph instead of the intended bar graph. Evidently, the student struggles to decide which graph type is suitable. Consequently, prospective teachers should note that the student’s incorrect answer likely stems from an inability to select the appropriate graph type, possibly due to insufficient knowledge about graph types and their proper applications or from inaccurate connections among related concepts. The student should recognize that selecting a suitable graph requires considering the problem situation, its context, and its purpose. However, the student finds it difficult to relate the context of the problem to the choice of graph. This difficulty may also arise because the student has previously used data solely to construct graphs and has not engaged in interpreting data. Prospective teachers might ask questions that prompt the student to inquire further, such as: *Why did you choose a line graph? Under what circumstances is it more appropriate to use a line graph? What does the value shown midway between any two years on the graph represent? Can you provide examples that illustrate situations in which a line graph is used? How would you interpret the graph when comparing English and French?* As corrective measures, prospective teachers should assign tasks that involve drawing line, bar, and pie charts and present example problem situations to guide students toward selecting the correct graph type.

In the items titled *Number of Train Services* and *Atakent–Pelitköy High School*, students are required to draw inferences from four different data sets. They are expected to interpret the data by considering measures of central tendency and dispersion and then make a prediction. However, the student supplies an incorrect answer. For the Number of Train Services item, prospective teachers might ask questions such as*: Why did you choose Train A? On what basis did you make this prediction? What if Train A completes the journey in ten hours? How do you view the run times of the trains? Which train or trains do you think have the shortest average run time? Can you calculate this? What information do you think you need? What is the mean? What is the range? How might these concepts inform our choice? Do you think these measures would assist in decision making? Could you compute an average run time for each train? Which trains show the greatest and smallest differences between run times? Can you use this information to decide? What might the sixth run time be for each train, and how would you determine it?* Similarly, in the *Atakent–Pelitköy High School* item, the student focuses on the larger numbers in each interval and, by choosing Atakent High School, draws an erroneous conclusion. The student should instead recognize that the table shows grouped data, that a mean cannot be calculated directly from a frequency distribution, and that the mean therefore cannot be used for inference. Secondly, the student should compare the number of students in each score interval with the total enrolment and make a decision by relating the score intervals to the overall success rates of the schools. Questions that might help the student notice the error include: *How did you decide that Atakent High School is preferred by many students? Do you think there is a relationship between high scores and the preferred school? If so, can you explain it? Can you represent the tabulated data with a graph? Which type of graph would you choose and why? Would your decision change if the score intervals were displayed, for example, as a bar graph? In that bar graph, which school has more students in the higher score intervals, and how would you interpret this? Has your decision changed? Why? Can you explain your decision? Can you suggest an alternative method to graphing for making a decision?* Proposed remedial strategies for prospective teachers might include *having students examine a data set, graph, and table to make decisions and formulate future predictions, using real life news texts with pictorial data from various sources selected by the students, and encouraging students to represent the tabulated data as a bar graph to facilitate correct inferences.*
