# Supplementary material for: Examining the infographic design instructional process in terms of prospective mathematics teachers’ infographic design proficiency, self‑efficacy, and abilities in evaluating student errors: A model proposal
Source: PLoS One. 2026 Apr 17;21(4):e0341380. doi: 10.1371/journal.pone.0341380 (PMC13089900; doi:10.1371/journal.pone.0341380)
Supplement: Attachment — Submitted filename: Response to Reviewers.docx [file pone.0341380.s005.docx]

**Response to Reviewers and Journal Requirements**

**Manuscript Title:** Examining the infographic design instructional process in terms of prospective mathematics teachers’ infographic design proficiency, self‑efficacy, and abilities in evaluating student errors: A model proposal

**Manuscript ID:** PONE-D-25-53363R1

| **Reviewer 1** | **Responses** |
| --- | --- |
| After carefully reviewing the manuscript entitled: “Examining the infographic design instructional process in terms of prospective mathematics teachers’ infographic design proficiency, self‑efficacy, and abilities in evaluating student errors: A model proposal” submitted by Neslihan Usta د, Ali Özkaya, Gözdegül Arık Karamık and Yusuf Akın, I find that the study addresses an important and contemporary research topic that is well aligned with the aims and scope of the Journal. The manuscript demonstrates originality, clearly defined objectives, and an appropriate methodological framework. The authors employed a research methodology suitable for the nature of the study, and the findings show logical consistency with the research questions and objectives. The results are adequately analyzed and supported by relevant and properly cited scholarly sources. Furthermore, the manuscript is well organized, clearly presented, and written in sound academic English. Based on the above considerations, I recommend acceptance of the manuscript for publication yours sincerely, | Thank you for your feedback. |
| **Reviewer 2** |  |
| First, the reliance on a single-group pre-test–post-test quasi-experimental design limits the ability to draw strong causal inferences. Although the authors acknowledge this limitation, it would be beneficial to discuss more explicitly how potential threats to internal validity (e.g., maturation, testing effects) may have influenced the results and how future studies could address these issues through control or comparison groups. | We added the following sentence Based on the limitations of the model used, explanations were provided regarding the effects of internal and external validity factors on the study, and suggestions were made for future research. (p. 32, 33 – lines 754-789) |
| Second, while the Data Availability Statement explains restrictions related to participant consent and confidentiality, the manuscript does not provide access to anonymized datasets or example data files underlying the quantitative analyses. This only partially meets PLOS ONE’s data-sharing requirements. The authors are encouraged to clarify whether de-identified or aggregated datasets, scoring rubrics, or sample coded data can be shared as Supporting Information to enhance transparency and reproducibility. | <https://doi.org/10.6084/m9.figshare.31283989>  The DOI containing the data related to the study has been added to the system. |
| Third, although the manuscript is generally well written and intelligible, minor language and stylistic issues remain, including occasional long or repetitive sentences and minor grammatical inconsistencies. Careful proofreading is recommended to improve clarity and conciseness, particularly in the Results and Discussion sections. | The necessary revisions have been made and the Results and Discussion section has been restructured.  In response to these suggestions, we have revised the relevant parts of the text. The changes have been made throughout the whole manuscript. Multiple sections were edited to improve clarity, consistency, and readability (lines: 19, 34, 39, 55, 58, 73, 93, 110, 116, 122, 128, 136, 137, 172, 179, 191, 192, 204, 209, 213, 241, 248, 254, 308, 326, 327, 334, 337, 340, 352, 359, 392, 402, 403, 412, 413, 417, 418, 421, 437, 438, 489, 492, 493, 500, 502, 503, 504, 505, 507, 521, 523, 524, 525, 526, 529, 540, 541, 542, 552, 553, 556, 558, 559, 560, 561, 573, 580, 593, 631, 632, 633, 656, 664, 679, 686, 706, 709, 720, 730, 731, 734, 737, 739, 740, 744, 746, 791, 792, 802, 803, 804, 805, 806.)  These revisions include corrections related to punctuation (e.g., periods, commas, and hyphen usage), as well as terminology adjustments. In several instances, the term “teacher” was replaced with the more accurate term “prospective teacher.” Similarly, some occurrences of “solution” were revised to “solution proposals” to better reflect the intended meaning. |
| **Journal Requirements** |  |
| 1. When submitting your revision, we need you to address these additional requirements.    Please ensure that your manuscript meets PLOS ONE's style requirements, including those for file naming. The PLOS ONE style templates can be found at | We checked the name of files. |
| 2. We note that you have indicated that there are restrictions to data sharing for this study. For studies involving human research participant data or other sensitive data, we encourage authors to share de-identified or anonymized data. However, when data cannot be publicly shared for ethical reasons, we allow authors to make their data sets available upon request | The DOI containing the data related to the study has been added to the system. |
| 3. Please include your full ethics statement in the ‘Methods’ section of your manuscript file. In your statement, please include the full name of the IRB or ethics committee who approved or waived your study, as well as whether or not you obtained informed written or verbal consent. If consent was waived for your study, please include this information in your statement as well. | Ethical considerations have been added to methods section. (p. 11, lines 269-275) |
| 4. Please remove your figures from within your manuscript file, leaving only the individual TIFF/EPS image files, uploaded separately. These will be automatically included in the reviewers’ PDF. | We have removed the figures from the article text. |
| 5. Please upload a new copy of Figures 4, 5 and 6, as the detail is not clear. | We uploaded figures.  Figures have been reorganized to improve clarity and visual readability. In the previous version, some figures contained multiple infographic examples. To ensure that each infographic is clearly visible and interpretable, these composite figures were divided into separate figures. Accordingly, the original figures were restructured and renumbered. |
|  | **The original figure captions and numbers are shown on the left column, and the revised figure captions and numbers are shown on the right column.**  **The updated figures are presented as follows:** |
| Fig 3. Infographics on Tangram and Mangala Designed on a Free Topic During the Preliminary Phase | Fig 3. Infographic on Tangram designed on a free topic during the preliminary phase.  Fig 4. Infographic on Mangala designed on a free topic during the preliminary phase (Certain images have been replaced to comply with copyright requirements.) |
| Fig 4. Chess Themed Infographics Designed on a Free Topic During the Preliminary Phase | Fig 5. Example of a chess-themed infographic designed on a free topic during the preliminary phase.  Fig 6. Another example of a chess-themed infographic designed on a free topic during the preliminary phase. |
| Fig 5. Infographics Entitled Bread Waste and Women and Men Designed at the Beginning of the Main Intervention | Fig 7. Infographic entitled Bread Waste designed at the beginning of the main intervention.  Fig 8. Infographic entitled Women and Men designed at the beginning of the main intervention. |
| Fig 6. Infographics Entitled Turkey’s Population Data and Mathematics Achievement Designed at the End of the Main Intervention | Fig 9. Infographic entitled Türkiye’s Population Data designed at the end of the main intervention.  Fig 10. Infographic entitled Mathematics Achievement designed at the end of the main intervention. |
| Fig 7. Infographics Entitled Youth Population in Turkey and Turkey’s Music-Listening Habits Designed at the End of the Main Intervention | Fig 11. Infographic entitled Youth Population in Türkiye designed at the end of the main intervention.  Fig 12. Infographic entitled Türkiye’s Music-Listening Habits designed at the end of the main intervention. |
| 6. We note that Figures 3, 4 and 7, in your submission contain copyrighted images. All PLOS content is published under the Creative Commons Attribution License (CC BY 4.0), which means that the manuscript, images, and Supporting Information files will be freely available online, and any third party is permitted to access, download, copy, distribute, and use these materials in any way, even commercially, with proper attribution | We checked figures. We also uploaded copyright transfer and usage permission form of infographic creators. |
| 7. We note that Figure 7, in your submission contain map images which may be copyrighted. All PLOS content is published under the Creative Commons Attribution License (CC BY 4.0), which means that the manuscript, images, and Supporting Information files will be freely available online, and any third party is permitted to access, download, copy, distribute, and use these materials in any way, even commercially, with proper attribution. For these reasons, we cannot publish previously copyrighted maps or satellite images created using proprietary data, such as Google software (Google Maps, Street View, and Earth). For more information, see our copyright guidelines: <http://journals.plos.org/plosone/s/licenses-and-copyright>. | We checked figure 7 (now figure 11) . |
| 8. If the reviewer comments include a recommendation to cite specific previously published works, please review and evaluate these publications to determine whether they are relevant and should be cited. There is no requirement to cite these works unless the editor has indicated otherwise. | There were no citation requests in the reviewer comments. |
| 9. Please review your reference list to ensure that it is complete and correct. If you have cited papers that have been retracted, please include the rationale for doing so in the manuscript text or remove these references and replace them with relevant current references. Any changes to the reference list should be mentioned in the rebuttal letter that accompanies your revised manuscript. If you need to cite a retracted article, indicate the article’s retracted status in the References list and also include a citation and full reference for the retraction notice. | Added to the references:  71. Fraenkel JR, Wallen NE. How to design and evaluate research in education. New York, NY: McGraw-Hill; 1990.  72. Karasar N. Bilimsel araştırma yöntemi [Scientific research method]. 14th ed. Ankara: Nobel Publication Distribution; 2005.  References 8, 9, and 10 have been revised and corrected. The source that remained in a different language has been adjusted, and the other two references have been updated as proper Internet sources in accordance with the journal’s Vancouver referencing style.  We have also revised the references by converting the previously provided HTTP links into proper DOI-based citations, when available, in accordance with the Vancouver referencing style. |
